# Fragment-Based and Structural Investigation for Discovery of JNK3 Inhibitors

**DOI:** 10.3390/pharmaceutics14091900

**Published:** 2022-09-08

**Authors:** Men Thi Hoai Duong, Hee-Chul Ahn

**Affiliations:** College of Pharmacy, Dongguk University-Seoul, Goyang, Gyeonggi 10326, Korea

**Keywords:** JNK3, fragment, saturation transfer difference NMR, X-ray crystallography

## Abstract

The c-Jun N-terminal kinases (JNKs) are members of the mitogen-activated protein kinase (MAPK) family and are related to cell proliferation, gene expression, and cell death. JNK isoform 3 (JNK3) is an important therapeutic target in varieties of pathological conditions including cancers and neuronal death. There is no approved drug targeting JNKs. To discover chemical inhibitors of JNK3, virtual fragment screening, the saturation transfer difference (STD) NMR, in vitro kinase assay, and X-ray crystallography were employed. A total of 27 fragments from the virtually selected 494 compounds were identified as initial hits via STD NMR and some compounds showed the inhibition of the activity of JNK3 in vitro. The structures of JNK3 with a fragment and a potent inhibitor were determined by X-ray crystallography. The fragment and inhibitor shared a common JNK3-binding feature. The result shows that fragment screening by NMR spectroscopy is a very efficient method to screen JNK3 binders and the structure of JNK3-inhibitor complex can be used to design and develop more potent inhibitors.

## 1. Introduction

JNKs are the serine/threonine kinases and one of the members of the mitogen-activated protein kinases [1,2]. JNK phosphorylates several substrates, such as c-Jun and activating transcription factor 2 in response to environmental stress and pro-inflammatory cytokines [2]. JNKs are involved in varieties of physiological processes including neuronal function, immune activity, and embryonic development [3,4]. Thus, it is implicated that the JNK pathway is related to various pathological conditions, including neurodegenerative diseases, cancer, and inflammation [5,6,7]. Since the JNK signaling pathway is involved in numerous inflammatory diseases, the inhibition of JNK signaling could decrease the expression of pro-inflammatory cytokines [8,9]. In cancer, some isoforms of JNKs are pro-oncogenic [10], whereas others act as tumor suppressors [11,12]. These findings imply the necessity of isoform-specific JNK inhibitors in the development of the cancer therapeutics.

Particularly, JNK3 is almost exclusively expressed in the brain, with very low levels in the kidneys and the testis, while JNK1 and JNK2 are widely expressed in a variety of tissues [3]. The level of the phosphorylated JNKs increased in postmortem brain tissue samples of Alzheimer’s disease (AD) patients [13] and was connected to the rate of cognitive decline [14]. Several studies demonstrated that JNKs are activated in Parkinson’s disease (PD) mouse models [15,16,17] and dopaminergic neurons were protected from apoptosis due to the inhibition of JNK by its specific inhibitor, SP600125 [18]. This evidence supports JNK3 as a promising target for the treatment of neurodegenerative diseases.

There have been great efforts to design and develop pan- and isoform-specific JNK inhibitors. Particularly, the structure determination of the JNK-inhibitor complex aided the pursuit of the development of novel and potent JNK inhibitors. Recently, we reviewed most of the available structures of JNK-inhibitor complexes [19]. Several hinge-binding scaffolds, such as aminopyrimidine, (iso)quinoline, quinazoline, and other scaffolds similar to purine, were frequently utilized for the development of ATP-competitive kinase inhibitors and 63 ATP competitive kinase inhibitors were FDA-approved till in the year 2021 [20,21]. Even though great efforts were paid to develop JNK inhibitors, there are no clinically available drug molecules as JNK therapeutics until now.

Fragment-based drug discovery (FBDD) has been an important paradigm for new drug discovery and is actively used in many pharmaceutical companies [22,23]. FBDD starts with the fragment screening in which weak binders to a target protein are selected and followed by creating lead compounds based on the fragment hits. Therefore, the screening methods generally rely on the biophysical methods to observe the weak interaction between the fragments and proteins. Those include the fluorescence-based method, NMR, surface plasmon resonance (SPR), isothermal titration calorimetry (ITC), mass spectroscopy, and X-ray crystallography. FBDD can be applied to the discovery of new drugs against most of the therapeutic targets, regardless of the types and characteristics of the proteins. Therefore, by using this method, several drugs have already entered the market, and many drug candidates are in clinical trials [24,25,26]. In discovering new kinase inhibitors, the fragment-based approaches have provided powerful insights into the selectivity enhancement and the lead optimization [27,28].

Compared with other methods, the NMR method has many advantages. The NMR spectroscopy is versatile and includes various methods of measurements to identify the weak binders to proteins. For example, STD NMR [29,30,31], relaxation filter experiments [32,33], and waterLOGSY [34] are commonly used for a ligand-based approach. A protein-based method is also utilized to identify protein binders, where the chemical shifts of the backbond -HNs of a target protein in two-dimensional (2D) ^1^H-^15^N heteronuclear single quantum correlation (HSQC) spectrum are monitored in the absence and presence of fragments. If the backbone chemical shift assignments are completed, the ligand binding “hot spot” can be identified without the knowledge of the detailed three-dimensional (3D) structure of the target. This provides an important information to create more potent compounds based on the fragments [22,23]. Even though the NMR methods are some of the most popular tools in FBDD campaign, it is not a stand-alone method and should be used complementarily with other methods to reduce the false positives, such as with X-ray crystallography for the identification of the exact binding mode and the assessment of structural novelty of the binding fragment. In this work, we present a collection of a fragment library by virtual screening, STD NMR spectroscopy, in vitro kinase assay, and the 3D structures of JNK3 in complex with fragments and an inhibitor, cyclopropyl[(3R)-3-({4-[6-hydroxy-2-(naphthalen-2-yl)-1H-benzimidazol-1-yl]pyrimidin-2-yl}amino)piperidine-1-yl]methanone, which previously showed a strong binding affinity to JNK3 and presented a potency of the cell protective effect in neuronal cell apoptosis [35]. In the structural investigation, we found a common JNK3-binding feature of the fragment and inhibitor, which occurred in the solvent-exposed region near the ATP binding site. We suppose this interaction potentiates the affinity of a chemical binder to JNK3 and could be utilized to design novel JNK3 inhibitors.

## 2. Materials and Methods

### 2.1. Fragment Library

The collection of compounds for the fragment library was conducted by virtual screening with the known structure of JNK3 (PDB ID, 1JNK) [36]. The bound molecules such as AMP-PNP, magnesium ions, and water were removed for the docking simulation. Docking simulations were performed on the ATP binding site of JNK3 by using the software Autodock vina [37] with the standard protocol provided by developer. The chemicals for the docking simulation were collected virtually from the various vendors’ websites (Asinex, Winston-Salem, NC, USA; Maybridge, Waltham, NA, USA; ChemiDive, San Diego, CA, USA) with the molecular weight being less than 300 Da. A total of 15,000 compounds were screened, and top-scored molecules were chosen. Of those commercially available, 494 compounds were purchased from Asinex. The information of the fragments is provided in the Supplementary Files S1 (list) and S2 (structure data file, sdf).

### 2.2. Preparation of JNK3

The gene encoding the catalytic domain of human JNK3 which spans the amino acid residues from Ser40 to was amplified by PCR and inserted into the pET-15b expression vector between NcoI and BamHI restriction sites. The plasmid was transformed into the *Escherichia coli* strain BL21 (DE3) (Merck KGaA, Darmstadt, Germany). Cells were grown at 37 °C utill OD_600_ reached to ~0.6–0.7 and induced with a final concentration of 0.5 mM isopropyl-β-D-thiogalactopyranoside (IPTG) for 16–20 h at 18 °C. Cells were harvested by centrifugation 4000 rpm at 4 °C. The cell pellet was re-suspended in 20 mL lysis buffer (20 mM Hepes, pH = 7.0, 20 mM NaCl, 10% (*v*/*v*) glycerol, 2 mM dithiothreitol (DTT)). Cell membranes were disrupted by sonication on ice. The lysate was clarified by centrifugation at 18,000 rpm for 40 min. The supernatant was used for the following steps of purification.

Ion exchange chromatography using SP-Sepharose FF 16/10 (GE Healthcare, Chicago, IL, USA) and size-exclusion chromatography using HiLoad 16/600 Superdex 75 prep-grade (GE Healthcare) were applied sequentially to purify JNK3. The purity of JNK3 was more than 95% judged by SDS-PAGE. The purified JNK3 was collected and concentrated using an Amicon (Merck KGaA, Darmstadt, Germany) centrifugal filter with MWCO 10,000 to 10 mg/mL. The protein was frozen under liquid nitrogen and finally stored at −80 °C.

### 2.3. NMR Experiments

All NMR data were measured at 25 °C on Agilent DD2 600 MHz NMR spectrometer with TR^TM^ probe (Agilent, Santa Clara, CA, USA). The 494 compounds were respectively dissolved in _d6_-DMSO for the preparation of stock solutions. The stock was diluted into _d11-_Tris-HCl buffer (in D_2_O), pH 7.5 with the final concentration of 320 μM in 500 μL. The simple ^1^H NMR spectrum of each compound was recorded with the sweep widths, 8370.5 Hz, and the time-domain points, 32K, for all experiments. Eight compounds were mixed, and the mixture was put to a single NMR tube, followed by the ^1^H NMR measurement. The final concentration of a single compound was 160 μM.

The purified JNK3 was added to an NMR tube containing 8 compounds to the final concentration of 4 μM. In this way, a total of 62 NMR tubes containing 8 compounds (in the last tube, 6 compounds) and JNK were prepared. ^1^H and STD NMR spectra were recorded, respectively. For STD NMR, the reference spectrum was acquired with the off-resonance saturation at 30 ppm, and the saturation spectrum was acquired with the on-resonance saturation at 0.5 ppm. The duration of the saturations was 3 s for both on- and off-resonance experiments. All data were processed and analyzed with the software Mnova (Mestrelab Research, Santiago de Compostela, Spain).

### 2.4. Crystallization and Data Collection

The recombinant human JNK3 (10 mg/mL) solution containing 0.02% n-octyl-glucopyranoside was mixed with ATP to a final concentration of 2 mM and crystallized as described previously [38] with some modification. The JNK3-AMP crystal was obtained by vapor diffusion in hanging drops at 4 °C with a reservoir solution containing 16% PEG MME 550, 10% ethylene glycol, 0.1 M HEPES pH 7.25, and 10 mM TCEP. JNK3-fragment (or inhibitor) crystals were obtained by micro-seeding the JNK3-AMP crystal under the addition of the fragment (or inhibitor) to the crystallization solution. The inhibitor was cyclopropyl[(3R)-3-({4-[6-hydroxy-2-(naphthalen-2-yl)-1H-benzimidazol-1-yl]pyrimidin-2-yl}amino)piperidine-1-yl]methanone (inhibitor, here after), which bound very strongly to JNK3 (*K_d_*, 46 nM) [35]. The diffraction data were collected at the 7A and 5C beamline of Pohang Light Source, Korea. The raw data were processed with HKL2000 [39]. The crystals belonged to space group of P212121, and the asymmetric unit contained one monomer (Table 1).

### 2.5. Structure Determination and Refinement

The structures were solved by the molecular replacement method using the program PHASER [40] with the template model PDB ID 3OY1, one of the JNK3 structure in complex with a selective inhibitor [41]. A manual model was built using the program COOT [42], and the resulting models were refined with the program REFMAC5 [43]. The quality of the refined models was evaluated by the program MolProbity [44]. Data collection and refinement statistics are summarized in Table 1. All figures representing the structure were generated by the *PyMOL* molecular–graphics program (The PyMOL Molecular Graphics System, Schrödinger, LLC., New York, NY, USA) [45] and by UCSF Chimera program [46]. The atomic coordinate and structure factors have been deposited in the Protein Data Bank (http://www.rcsb.org) with accession number of 4KKH and 7YL1 for fragment- and inhibitor-bound JNK3, respectively.

**Table 1:** Data collection and refinement statistics.

Crystals	3A8	Inhibitor
PDB ID	7YL1	4KKH
Data collection		
Space group	P212121	P212121
Cell dimensions		
a, b, c (Å)	50.15, 71.98, 107.16	52.32, 71.50, 107.08
α, β, γ (°)	90, 90, 90	90, 90, 90
Wavelength (Å)	0.97951	0.97951
Resolution (Å) *	59.75–2.48 (2.55–2.49)	50–2.0 (2.03–2.00)
Redundancy *	13.3 (12.5)	13.5 (12.8)
Completeness (%) *	99.1 (99.4)	100 (100)
I/σ_I_ *	33.6 (3.7)	58.9 (9.4)
R_merge_ (%) ^a,^*	9.0 (35.3)	9.0 (44.2)
Refinement		
Resolution (Å)	36.61–2.49	37.45–2.0
No. of reflections	13,406	26,471
R_work/_R_free_ (%) ^b^	20.4/29.4	22.9/27.7
No. of atoms	2925	2942
Average B-factors	Protein/water/3A8 43.139/42.040/48.722	Protein/water/inhibitor 43.774/47.582/35.827
R.m.s. deviations		
Bond lengths (Å)	0.007	0.007
Bond angle (°)	1.157	1.109
Ramachandran plot		
Residues in favored/allowed/ disallowed regions (%) ^c^	93.9/5.5/0.6	97.4/2.6/0/0

* Values in the parentheses refer to the highest resolution shells. ^a^ *R*_merge_ = Σ*_i_*Σ*_i_*|*I*(*h*)*_i_*− <*I*(*h*) > |/Σ*_h_*Σ*_i_I*(*h*)*_i_*, where *I*(*h*) is the intensity of reflection *h*, Σ*_h_* is the sum over all reflections, and Σ*_i_* is the sum over *i* measurements of reflection *h*. ^b^ *R*_work_ = Σ||*F*_obs_| − |*F*_calc_||/Σ|*F*_obs_|, 5% of the data was set aside for *R*_free_ calculation. ^c^ Statistics according to MolProbity Ramachandran analysis [44].

### 2.6. In Vitro Kinase Assay

The in vitro kinase assay was carried out with ADP-Glo^TM^ Kinase assay kit (Promega, Madison, WI). The assay was prepared with the final concentration of 2 ng of active JNK3, 50 μM ATP, 100 μM fragments, 1X reaction buffer, and 0.04 μg/μL p38 substrate. The experiments were set up with three control experiments including without ATP, without active JNK3, and without compound.

The mixture was incubated for 1 h at room temperature. The incubated solution was then supplied with 50 μL ADP-Glo reagent, centrifuged with 6000 rpm at 26 °C for 30 s, and incubated for 40 min at room temperature. The samples were transferred to a 96-white plate and added kinase-detection reagent with the ratio of 1:1. After incubating for 40 min at room temperature, the luminescence signals were recorded using the GloMax^®^-Multi Microplate Reader (Promega, Madison, WI, USA). The kinase assays were duplicated for each fragment.

## 3. Results

### 3.1. Virtual Screening

To build a fragment library to screen potential JNK3 inhibitors, about 15,000 compounds with molecular weights less than 300 Da from the vendors Asinex, Maybridge, and ChemiDive were collected virtually. We performed structure-based virtual screening using the 3D structure of JNK3 (PDB ID, 3DA6) retrieved from Protein Data Bank (PDB) (http://www.rcsb.org). AutoDock Vina [37] was utilized for the molecular docking. The docking site of fragments was set at the ATP-binding site of JNK3. From the simulation, 494 compounds presenting docking scores less than −7 kcal/mol were chosen. The structure data file of 494 compounds is available in the Supplementary File S2.

### 3.2. Fragment Screening

First, the individual ^1^H NMR spectra of 494 compounds were measured and used as ‘reference spectra’ of the fragment library. Since we performed the NMR experiments in an aqueous buffer, the NMR spectra of almost 100 compounds were not able to be measured because of the poor solubility of those chemicals. To reduce the time of NMR measurements, we added eight compounds in a single NMR tube and recorded ^1^H NMR spectrum. Since the number of compounds in the library was 494, the number of 8 chemical mixtures in total were 62, where 61 contained 8 compounds, and the last tube contained 6 compounds.

**Figure 1:**
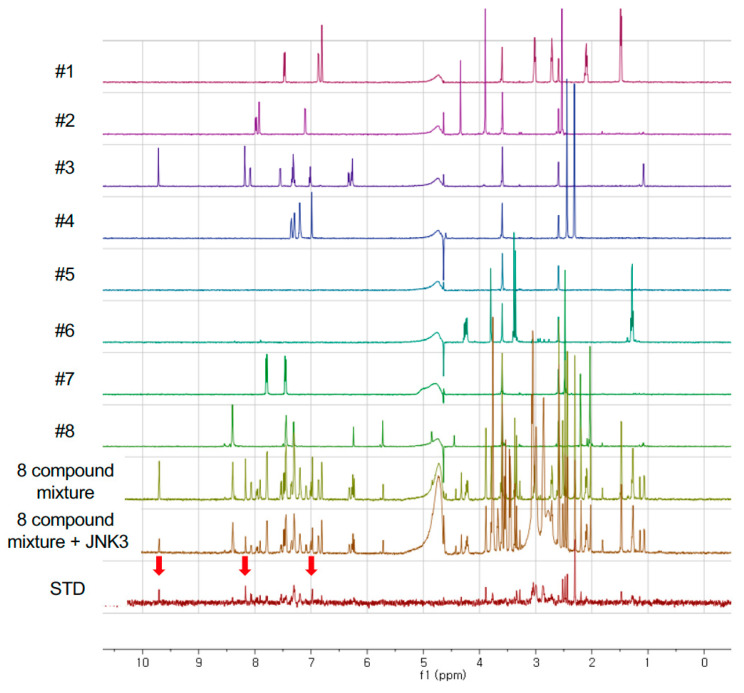
Reference NMR spectra of fragments and STD NMR spectrum. The spectra from #1 to #8 were the individual spectrum of fragment in a single NMR tube, respectively. STD NMR spectrum showed that compound #3 might bind to JNK3 (indicated by red arrow).

If there is no reaction or interaction between compounds in a single NMR tube, the NMR spectrum of the mixture is a simple sum of the spectra of each compound. In Figure 1, it is clear that the spectra of 8 compound mixtures is exactly the same of the overlay of individual spectrum of 8 compounds. When JNK3 was added to the 8 compound mixtures, the NMR spectrum was crowded in the region 2–4 ppm since the added protein solution contained some additives such as buffer ingredients, Hepes, and DTT; however most of the compound signals were clearly shown. In the STD NMR spectrum, we found several NMR signals which were the signals of possible JNK3-binding molecules. In this way, we found that compound #3 would have the possibility to bind to JNK3 (bottom of Figure 1).

Through STD NMR experiments, we chose 27 compounds which might bind to JNK3; thus, the hit rate was about 6.9% when we excluded the insoluble compounds. Some of the selected compounds shared a common chemical structure which is similar to purine (Table 2). Among them, aliphatic chains at R_1_ position and sulfur at R_2_ are in common.

### 3.3. In Vitro Kinase Assay

**Figure 2:**
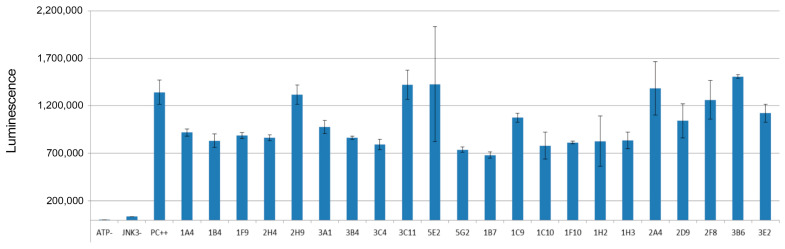
In vitro kinase assay and the inhibitory effects of selected fragments. PC++ stands for the positive control without any fragment molecules. The experiments were duplicated, and the errors were indicated.

The in vitro kinase assay was performed in the presence of the selected compounds to check whether those compounds were able to inhibit the catalytic activity of JNK3. For the fragments 2D5, 2D8, 3A8, 3B7, and 3B11, it was not possible to measure the inhibitory effects, possibly because of the solubility issue of those compounds in the reaction condition. The positive control without the treatment of any fragments showed significant luminescence since in ADP-Glo^TM^ assay ADP converted from ATP by active JNK3 was monitored by luminescence. Thus, if any fragmented molecules inhibited the enzymatic activity of JNK3, the production of ADP would be reduced, resulting in the decrease of luminescence signal. In Figure 2, the summary of the inhibitory effects of selected 22 fragments was provided. Some of those did not show any inhibitory effect against JNK3; however, several fragments including 1B7 showed about 40–50% inhibition of JNK3 activity at the fragment concentration of 100 μM.

### 3.4. Overall Structure of JNK3 in Complex with Fragment and Inhibitor

**Figure 3:**
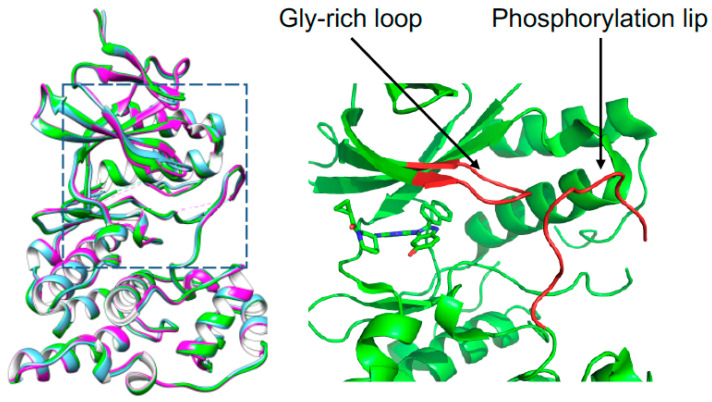
Overall structure of apo-(purple), fragment-bound (cyan), and inhibitor-bound (green) JNK3. The structural difference occurred at glycine-rich region and phosphorylation lip, which were enlarged on right and indicated. In the overlayed structures, the bound molecules were removed for the clarity of structural comparison.

We tried the crystallization of JNK3 with all the selected fragments by using the micro-seeding or fragment-soaking methods, but a limited number of crystals was acquired. Of those, only the structure with a fragment 3A8 (7-hexyl-3-methyl-8-sulfanylidene-9H-purine-2,6-dione) was determined, since other complex crystals gave rise to poor diffraction. Another crystal structure of human JNK3 in a complex with a potent inhibitor, cyclopro-pyl[(3R)-3-({4-[6-hydroxy-2-(naphthalen-2-yl)-1H-benzimidazol-1-yl]pyrimidin-2-yl}amino)piperidin-1-yl]methanone was also determined by molecular replacement. The Ramachandran plot of 3A8 bound JNK3 shows that 93.9% of non-glycine and non-proline residues are in the most favored regions; 5.5% of residues are in the additional allowed regions; and two residues, Arg212 and Asp381, in the disallowed regions. For the inhibitor-bound JNK3, those values were 97.4, 2.6, and 0%, respectively. The data and refinement statistics are summarized in Table 2. The structures of JNK3 were almost identical to the previously reported structures of JNK3. The root mean square (r. m. s.) deviation between the 3A8 bound JNK3 and AMP-PNP-bound JNK3 (PDB ID, 1JNK) was 0.392 Å for 343 Cα atom pairs and the r. m. s. deviation between the inhibitor-bound forms and AMP-PNP-bound JNK3 was 0.495 Å for 340 Cα atom pairs. The r. m. s. deviation of the two structures determined in this work was 0.533 Å for 345 Cα atom pairs (Figure 3). Very subtle differences were found in the glycine-rich region (Gly71-Ser-Gly-Ala-Gln-Gly-Ile-Val78) and the phosphorylation lip (Ser217-Thr226). In the inhibitor-bound structure, the glycine-rich loop and the phosphorylation lip moved little bit toward ATP-binding site.

### 3.5. Structure of JNK3 in Complex with Fragment 3A8

**Figure 4:**
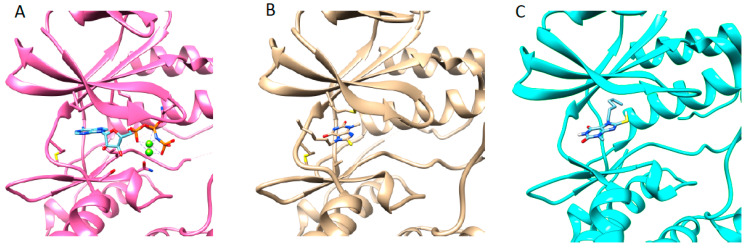
Structure of JNK3 in complex with AMP-PNP ((**A**), PDB ID, 1JNK) and fragment 3A8 (**B**), and docking simulated structure of JNK3 with 3A8 (**C**). Small molecules were presented by stick. In AMP-PNP bound structure, two magnesium ions were involved in the complex.

The ATP-binding site of JNK3 is located between N-terminal lobe (residues 45–149) and C-terminal lobe (residues 150–211 and 217–374) [36]. The fragment 3A8 bound to the ATP binding site of JNK3. Since 3A8 contained a similar structure to purine, the binding mode of 3A8 was thought to be similar to AMP-PNP. The 3A8 was positioned at the adenine binding site of AMP-PMP, but the orientations of the pyrine moiety of 3A8 and the adenine of AMP-PNP were different by almost 90° degrees (Figure 4A,B). 3A8 binding to JNK3 was completely opposite to the docking pose. In virtual screening, the lowest energy docking pose of 3A8 to JNK3 scored −7.0 kcal/mol and the hexyl chain penetrated deeply into the hydrophobic region of the ATP binding site (Figure 4C). The hexyl chain of 3A8 in JNK3 crystal structure protruded from the ATP binding site toward the surface of the protein. The hydrophobic interaction between the hexyl group of 3A8 with residues in β-strands above the ATP binding site might contribute to the binding of fragment to enzyme. 

### 3.6. Structure JNK3 in Complex with a Potent Inhibitor

**Figure 5:**
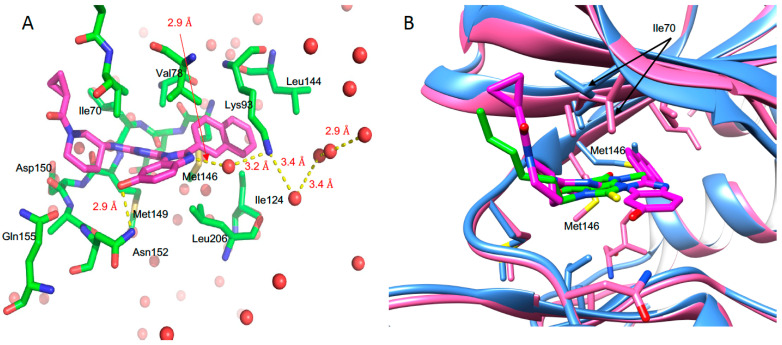
Structure of JNK3 in complexed with cyclopro-pyl[(3R)-3-({4-[6-hydroxy-2-(naphthalen-2-yl)-1H-benzimidazol-1-yl]pyrimidin-2-yl}amino)piperidin-1-yl]methanone (**A**). The residues involved in the interaction were indicated. Water molecules were indicated as red dots and hydrogen bonds as yellow broken lines. The lengths of hydrogen bonds are labeled in red. (**B**) Overlaid structure of JNK3 with 3A8 and inhibitor. Ile70 were indicated by arrow. Different positioning of Met146 in both structures are also indicated. JNK3 were colored blue for 3A8 bound form and pink for inhibitor bound form, respectively.

In the previous study, 1-Heteroaryl-2-aryl-1H-benzimidazole derivatives showed the inhibitory effect against JNK3 [35]. Cyclopropyl[(3R)-3-({4-[6-hydroxy-2-(naphthalen-2-yl)-1H-benzimidazol-1-yl]pyrimidin-2-yl}amino)piperidin-1-yl]methanone was one of the derivatives of those inhibitors with a high binding affinity (*K_d_*, 46 nM) and inhibitory potency (*IC_50_*, 27.7 nM) to JNK3 (Figure S1). In this work, we solved the crystal structure of JNK3 in complex with this inhibitor. The binding of inhibitor to JNK3 is illustrated in Figure 5A. 

The inhibitor resided on the ATP binding site of JNK3, and the naphthalene moiety penetrated deeply into the interior of ATP binding pocket, where ATP or AMP-PNP did not interact. The naphthalene ring moiety interacted with Met 146. A naphthalene ring-binding induced the movement of the side chain of Met146 (gatekeeper in JNK3), which had a different orientation in AMP-PNP bound JNK3 structures. The naphthalene ring moiety possessed hydrophobic interaction with Lys93, Met146, Ile124, Val78, Ala91, and Leu144. Met149 formed a hydrogen bond with a 2-aminopyrimidine moiety of the inhibitor, and Ile70 interacted with piperidine and 1-cyclopropyl moieties of inhibitor. The side chain of Ile70 was rotated for stable interaction with 1-cyclopropyl. Leu206, Asn152, Val78, and Ile70 interacted with the 6-Hydroxy-1H-benzimidazole moiety of inhibitor. The side chain of Asn152 formed a direct hydrogen bond with the hydroxyl group of the 6-Hydroxy-1H-benzimidazole moiety that formed water-mediated hydrogen bond with Lys93. Several water molecules were coordinated in the vicinity of Lys93, which interacted with the naphthalene ring moiety. 

When we compare the structures of JNK3 with a fragment and inhibitor, it was noticeable that the binding of 3A8 to JNK3 very much resembled the binding to the inhibitor, even though two chemicals did not share structural similarities, and the bicyclic rings of the two molecules were different. In the 3A8-bound structure, the 6-membered ring was outward from ATP binding site; however, in the inhibitor-bound one, 5-membered ring was outward and 6-membered ring inward. The common feature was the orientation and interaction of the hexyl group of 3A8 and the 1-cyclopropyl moiety of the inhibitor. Those two moieties protruded from the ATP binding site and presented hydrophobic interactions with the β-strand region of JNK3, where Ile 90 played a role. The importance of the hydrophobic interaction involving the 1-cyclopropyl moiety of the inhibitor on the binding and inhibitory activity to JNK3 are discussed below.

## 4. Discussion

We have conducted the in silico, in vitro, and structural investigations for the discovery of JNK3 inhibitors by adapting the fragment-based approach. 494-membered fragment library was constructed, and the library of NMR spectra was also built, even though about 20% of compounds did not give rise to the spectrum, because of the solubility problem. The hit rate from the screening from the STD NMR experiments was 6.9%, which is a relatively high value in a drug discovery campaign. In a drug repurposing project, we have shown that the longitudinal (T_1_) and transverse (T_2_) relaxation experiments and the STD NMR spectroscopy detected the JNK3 binding of azelastine, which has an original indication for anti-allergy and anti-inflammation effects and proven that azelastine binds to ATP binding pocket of JNK3 via competition STD experiment with AMP-PNP [47]. In this regard, we have shown that NMR spectroscopy is very efficient in drug discovery process, especially in initial fragment screening.

The kinase assay presented that most of the selected compounds had the inhibitory activity against JNK3, even though the effects were not potent. Since fragments are small and have simple structures, it is very hard to find potent molecules from a fragment-based approach. In this work, we just checked the inhibitory effects of the screened fragments to see whether those could inhibit the activity of JNK3 or not. The versatility of the fragment-based approach in drug discovery would be whether the screened fragment compounds could be used for designing and creating potent inhibitors. In this context, the structural information between fragments and the target proteins are very valuable. We have determined a structure of JNK3 in complex with one of the screened fragments as well as with a potent inhibitor. The fragment 3A8 had limited water solubility, thus, it was impossible to run the kinase assay. However, in the crystallization, certain additives such as 0.02% n-octyl-glucopyranoside might enhance the solubility of 3A8 in an aqueous solution. The 3A8 shared structural similarities to the adenine of ATP; however, the binding pattern was completely different from the adenine moiety of ATP. The hydrophobic hexyl group of 3A8 protruded from the ATP binding site and gave rise to additional interactions with the glycine-rich region of JNK3. We focused on the hydrophobic interaction to discover an isoform-specific JNK3 inhibitors. Every kinase shares a common ATP binding mode, which hampers the discovery of the specific kinase inhibitor, especially ATP-competitive inhibitors. For the discovery of potent and selective ATP-competitive JNK inhibitors, targeting hydrophobic interactions in combination with the interaction on ATP-binding sites was suggested [38]. Those interactions were ones with solvent-exposed hydrophobic regions, including a glycine-rich loop and another with deep hydrophobic region where the naphthalene moiety of inhibitor in this work penetrated. In the previous work, among 1-Heteroaryl-2-aryl-1H-benzimidazole derivatives, the inhibitor cyclopropyl[(3R)-3-({4-[6-hydroxy-2-(naphthalen-2-yl)-1H-benzimidazol-1-yl]pyrimidin-2-yl}amino)piperidin-1-yl]methanone showed the strongest binding affinity to JNK3. The binding affinity of this inhibitor was 60-fold stronger than the derivatives without 1-cyclopropyl moiety [35]. We performed the measurement of the inhibitory activity of the 1-Heteroaryl-2-aryl-1H-benzimidazole derivatives (Figure S1). The compound with the 1-cyclopropyl moiety (inhibitor in this work) showed the *IC_50_* of 27 nM, whereas the compound without the moiety showed the *IC_50_* of 1.41 mM, which coincides very well with previous binding experiments. When the naphthalene moiety was replaced by the dichlorophenyl moiety, the *IC_50_* increased 178-fold. We confirmed experimentally that the importance of targeting hydrophobic interactions in discovering JNK inhibitors. We suppose that those interactions would be related to the selectivity of JNK inhibitors. In Figure S2, the inhibitory activity of cyclopropyl[(3R)-3-({4-[6-hydroxy-2-(naphthalen-2-yl)-1H-benzimidazol-1-yl]pyrimidin-2-yl}amino)piperidin-1-yl]methanone against several MAPKs is shown. The inhibitor in this work had potency against JNK3, JNK1, p38α, and p38β, and showed none or little inhibitory effects on p38γ, and p38δ., ERK, and GSK. We suppose that the hydrophobic interaction mentioned above would provide the selectivity. Thus, we suggest that keeping this hydrophobic interaction is very important for the discovery of novel and potent JNK3 inhibitors.

## 5. Conclusions

We presented the fragment-based approach in discovering JNK3 kinase with the structural investigation. We showed that virtual screening and in vitro assay supported by the NMR-based fragment screening were very efficient in early drug-discovery projects. In the structural investigation, we have identified the hydrophobic interaction between a fragment and the enzyme and presented the importance of this interaction in the potency and selectivity of the JNK3 inhibitors. We believe that this work provides valuable insight in designing lead compounds based on the fragment in the discovery of kinase inhibitors.

## Figures and Tables

**Table 2:** Compounds sharing a common structure.

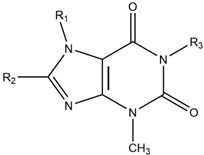			
Code	R_1_	R_2_	R_3_
1A4	-CH_3_	-SCH_2_COOH	-H
2H4	-CH_2_-CH=CH_2_	-SCH_2_COOH	-H
1F9	-CH_3_	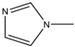	-CH_3_
2D5	-CH_2_-CH(CH_3_)_2_	-NH-NH_2_	-H
2D8	-CH_2_-CH=CH-CH_3_	-SH	-H
3A1	-CH_3_	-SH	-H
3A8	-CH_2_-CH_2_- CH_2_-CH_2_-CH_2_-CH_3_	-SH	-H
3B11	-CH_2_-CO-CH_3_	-H	-CH_3_
3B7	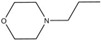	-H	-H
1C9	-CH(CH_3_)_2_	-S-CH_2_-CO-NH_2_	-H
2A4	-CH_2_-CH_2_-CH_3_	-S-CH_2_-CO-NH_2_	-H

## Data Availability

Not applicable.

## Supplementary Materials

The following supporting information can be downloaded at: https://www.mdpi.com/article/10.3390/pharmaceutics14091900/s1, Figure S1: Structures and inhibitory activities of 1-Heteroaryl-2-aryl-1H-benzimidazole derivatives; Figure S2: Inhibitory activity of cyclopro-pyl[(3R)-3-({4-[6-hydroxy-2-(naphthalen-2-yl)-1H-benzimidazol-1-yl]pyrimidin-2-yl}amino)piperidin-1-yl]methanone against various kinases; File S1: list_fragments.xlsx; File S2: SDF_fragments.sdf.
