# Supplementary material for: Fragment-Based and Structural Investigation for Discovery of JNK3 Inhibitors
Source: Pharmaceutics. 2022 Sep 8;14(9):1900. doi: 10.3390/pharmaceutics14091900 (PMC9501523; doi:10.3390/pharmaceutics14091900)
Supplement: Supplementary file 1 [file pharmaceutics-14-01900-s001.zip › pharmaceutics-1866392_supplementary.pdf]

Supplementary Figures for

Fragment-based and structural investigation for discovery of JNK3 inhibitors

Men Thi Hoai Duong and Hee-Chul Ahn *

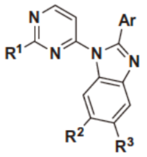

Comp	R1	R2	R3	Ar	IC_{50} (μM)
14	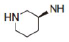	H	H	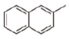	1.41 ± 0.40
15d	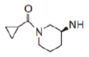	H	H	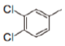	4.81 ± 1.19
16f	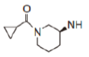	OH	H	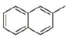	0.027 ± 0.004
SP600125	-	-	-	-	0.215 ± 0.065

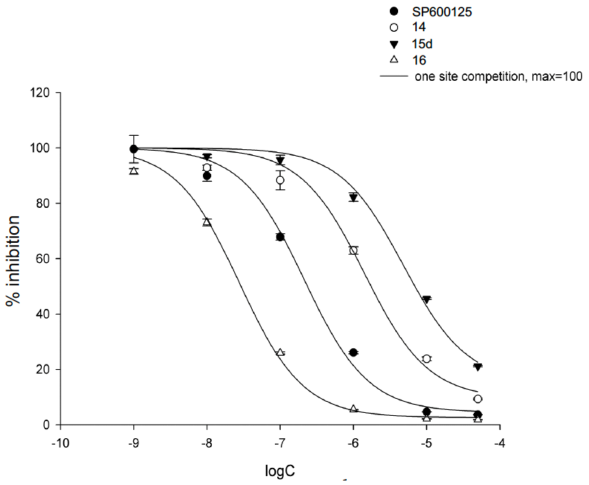

Figure S1. Structures and inhibitory activities of 1-Heteroaryl-2-aryl-1H-benzimidazole derivatives. The numbers of compounds were adapted from the literature [1]. SP600125 is a known JNK inhibitor. The measurements were triplicated and the data were fit to one site competition model in the program SigmaPlot.

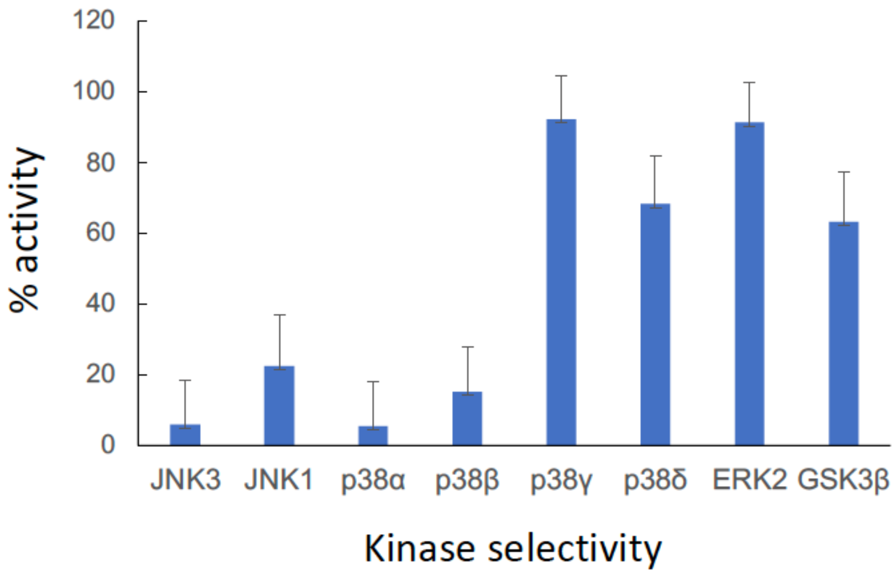

Figure S2. Inhibitory activity of cyclopro-pyl[(3R)-3-({4-[6-hydroxy-2-(naphthalen-2-yl)-1H-benzimidazol-1-yl]pyrimidin-2-yl}amino)piperidin-1-yl]methanone against various kinases. The kinases were provided by Promega (Madison, WI) and the assay was performed by the method described in the manuscript. All experiments were triplicated.

Reference

1. Kim, M.H.; Lee, J.; Jung, K.; Kim, M.; Park, Y.J.; Ahn, H.; Kwon, Y.H.; Hah, J.M. Syntheses and biological evaluation of 1-heteroaryl-2-aryl-1H-benzimidazole derivatives as c-Jun N-terminal kinase inhibitors with neuroprotective effects. *Bioorg Med Chem* **2013**, *21*, 2271–2285. https://doi.org/10.1016/j.bmc.2013.02.021.
